# Childhood obesity: The threatening apprentice of the adiposity empire

**DOI:** 10.1007/s11154-025-09959-4

**Published:** 2025-04-07

**Authors:** J. Karina Zapata, Javier Gómez-Ambrosi, Gema Frühbeck

**Affiliations:** 1 Department of Endocrinology and Nutrition, Clínica Universidad de Navarra, Pamplona, Spain; 2 Metabolic Research Laboratory, Clínica Universidad de Navarra, Pamplona, Spain; 3 Centro de Investigación Biomédica en Red-Fisiopatología de la Obesidad y Nutrición (CIBEROBN), Instituto de Salud Carlos III, Pamplona, Spain; 4 Instituto de Investigación Sanitaria de Navarra (IdiSNA), Pamplona, Spain

**Keywords:** Adiposity-based chronic disease, BMI, Body composition, Adipose tissue, Energy expenditure, Hormones, Comorbidities

## Abstract

Childhood obesity is a global health problem, with its prevalence having tripled since 1975. The increase in its prevalence has been predominantly in developing countries, but also in those with high economic status. Nowadays, there are multiple obesity definitions, however, one of the most accurate is the one which defines obesity as the accumulation of excessive body adiposity and not as an body weight excess. Nevertheless, the body mass index (BMI) is the most frequently used tool for its classification, according to the cut-off points established by the Center for Disease Control and World Health Organization tables. In children and adolescents an adiposity excess is related to the appearance of cardiovascular disease in adulthood and with many comorbidities such as metabolic syndrome, insulin resistance, type 2 diabetes, hypertension and metabolic dysfunction-associated steatotic liver disease, among others. Currently, there is still controversy about which is the ideal indicator for measuring overweight and obesity. BMI is still used as a standardized measure but may miss cases in which body composition is pathological despite a BMI within the normal-weight category. An adequate knowledge of the impact on health of dysfunctional adiposity as well as its accurate diagnosis will allow health professionals to address this condition in a more precise and comprehensive manner, and substantially improve the associated cardiometabolic risk and prognosis.

## Introduction

Childhood obesity is a global health problem, with an increase in its prevalence predominantly in developing countries, but also in those with high economic status [1, 2]. Noteworthy, childhood obesity has a high probability of continuing throughout adult life, being related to greater social stigma, cardiometabolic mortality, and premature death [3, 4].

## Definition

One of the most accurate obesity definitions is the accumulation of excessive body adiposity and not an excess body weight. However, body mass index (BMI) is the most frequently used tool for its classification. The BMI presents dynamic changes in childhood and adolescence, especially during puberty [5, 6]. For this reason, BMI percentiles are adjusted according to age and sex, categorizing children between the 5th and 85th percentile as normal weight, in the overweight category those with a BMI greater than or equal to the 85th percentile, and in the obesity range those with a BMI equal to or greater than the 95th percentile. These cut-off points are established by the United States Center for Disease control (CDC), for children over two years old, while for children under 2 years of age, the weight assessment must be done with the World Health Organization (WHO) tables [7, 8]. According to these reference curves from 2006/2007, the definition of childhood obesity is as follows:

- Children under 5 years. *Overweight:* weight for height more than 2 standard deviations above the median established in childhood growth patterns. *Obesity:* weight for height more than 3 standard deviations above the median established in childhood growth patterns [9].
- Children between 5–19 years. *Overweight:* BMI for age more than 1 standard deviation above the median established in childhood growth patterns. *Obesity:* greater than 2 standard deviations above the median established in childhood growth patterns [9].

The definition for severe obesity is derived from the CDC Growth Charts [10]. Noteworthy, severe obesity is increasing its prevalence in the pediatric population [8–10], among girls between 6–11 years and boys from 12 to 19 years [10]. Severe obesity encompasses a BMI greater than or equal to 120% of the 95th percentile or a BMI ≥ 35 kg/m^2^ (grade II obesity) and a BMI greater than or equal to 140% of the 95th percentile (grade III obesity) according to the American Heart Association.

**Fig. 1:**
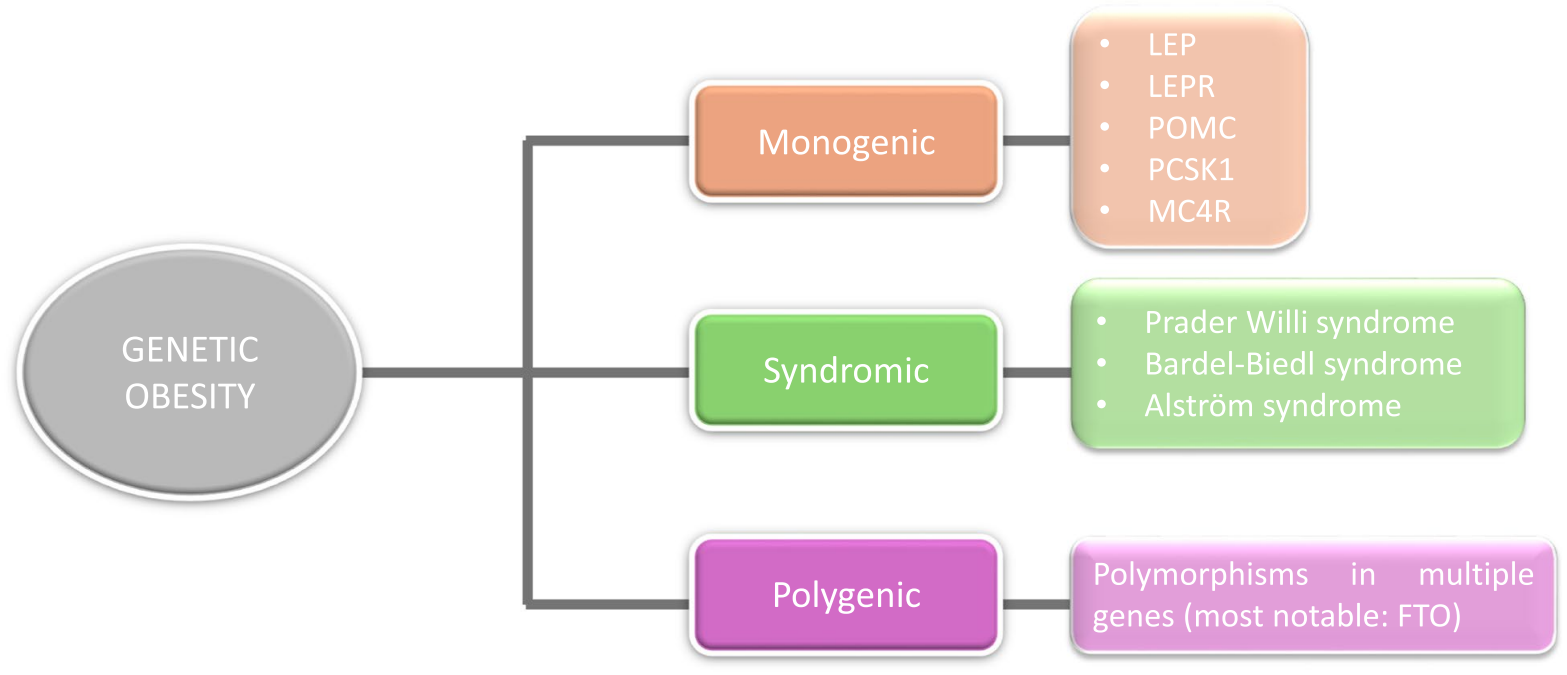
Genetic obesity classification. There are three main presentations of obesity associated to genetic causes: monogenic, syndromic, and polygenic obesity. Monogenic obesity occurs at early ages, and it is a rare and severe form caused by a single gene mutation. Syndromic obesity involves development abnormalities, neurodevelopmental deficits, and dysmorphic features. Polygenic obesity, known as common obesity, is triggered by the simultaneous presence of defects in multiple genes. Some representative examples are shown in the present figure. FTO: fat mass and obesity-associated protein, LERP: leptin receptor, MCR4: melanocortin receptor, PCSK1: proprotein convertase Subtilisin/Kexin type 1, POMC: proopiomelanocortin

Obesity is considered a chronic low-grade inflammation [11, 12]. The pathogenesis involves energy regulation, hunger, and physical activity, in association with genetic and environmental factors, socioeconomic status and health systems efficiency [12, 13]. Obesity is classified into 3 main groups based on genetic contribution (Fig. 1). *Monogenic* obesity is a rare and severe form caused by a single gene mutation. Affected genes oversee the appetite and satiety regulation at the central nervous system level, especially in the hypothalamic leptin-melanocortin pathway [14]. *Syndromic obesity* is accompanied by early onset, short stature, dysmorphic features, organ-specific developmental abnormalities, and neurodevelopmental deficits [15, 16]. *Polygenic* obesity is triggered by the simultaneous presence of multiple genes that favor a greater caloric intake [17], increasing appetite and body fat storage, and reducing satiety and overeating control [18].

Obesity is the consequence of a multifactorial disproportion between food intake and energy expenditure [19]. Energy intake is stimulated by homeostatic and hedonic processes. Homeostatic processes produce sensations of hunger and satiety and are regulated by sensory systems related to the gut-brain axis, modulating appetite satiation (fullness after finishing eating) and satiety (postprandial processes that establish the appetite recurrence) [20]. Environmental signals related with food intake as well as seeing, smelling, or tasting food trigger multiple physiological processes as production of saliva and gastrointestinal enzymes, and release hormones such as insulin [21]. Decreased energy stores produce changes in metabolism and food intake, promoting weight gain and maintenance [19].

**Fig. 2:**
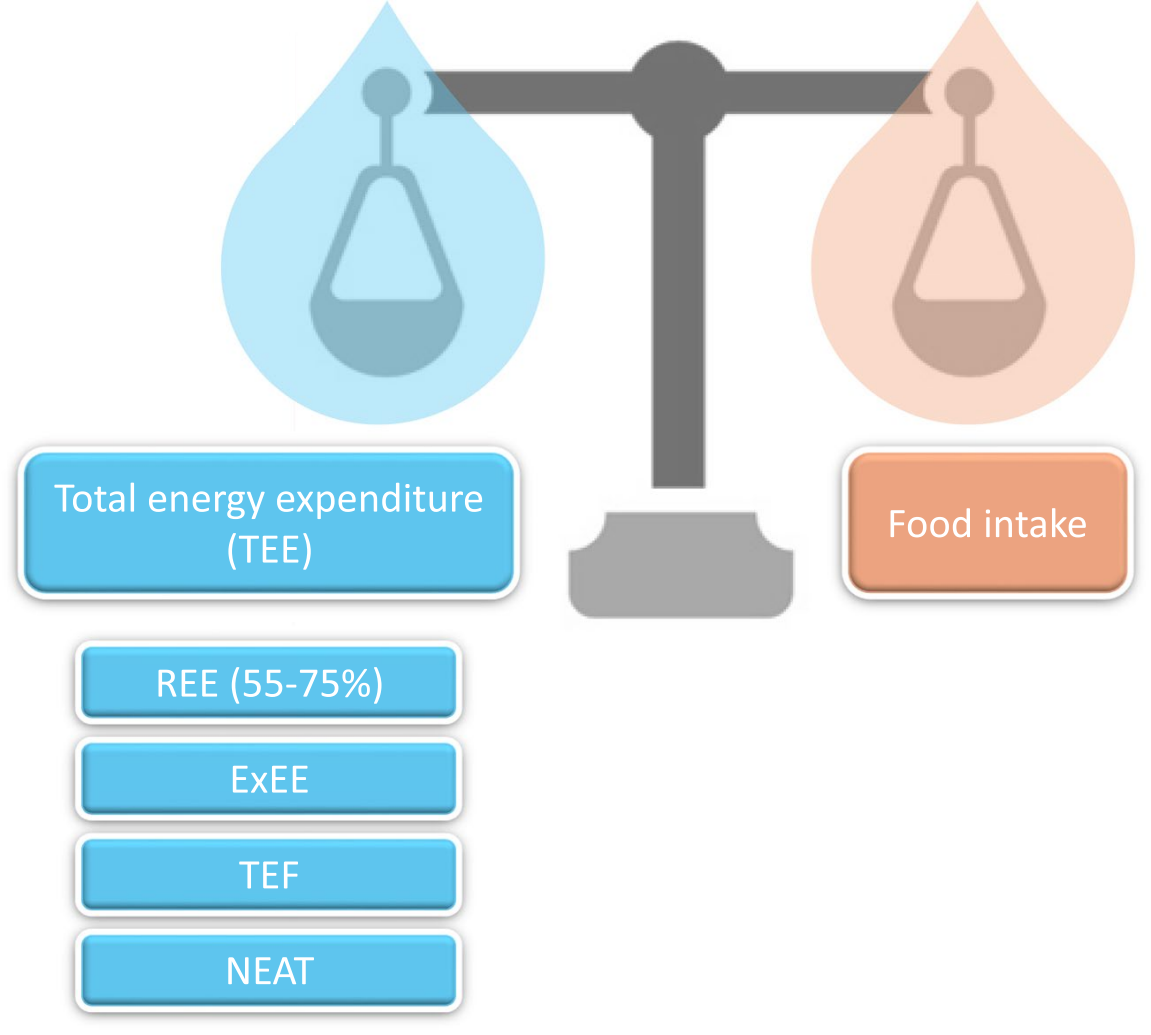
Energy homeostasis. Regulatory mechanisms involved in the obesity development may affect energy intake and energy expenditure. The total daily energy expenditure (TEE) is made up of the sum of calories that the human body burns in 24 h, and results from resting energy expenditure (REE), diet-induced thermogenesis or thermic effect of feeding (TEF), exercise energy expenditure (ExEE) and non-exercise activity thermogenesis (NEAT). REE represents the minimal amount of energy that is required to maintain all the body functions at the basal state and contributes about 55–75% of the TEE, TEF is related to the amount of energy required for the digestion, absorption, usage and storage of nutrients after eating and represents 5–15% of the TEE, ExEE is the most variable component and is defined as the energy burned during physical activity and is about 15–30% of the TEE, and NEAT involves the energy expenditure that is not typically consider and results from spontaneous physical activity that is not the result of voluntary exercise, as the energy expended in maintain and changing posture and other activities of daily living that requires low energy consumption, NEAT is responsible for 6–10% of the TEE. ExEE: exercise energy expenditure, NEAT: non exercise activity thermogenesis, REE: resting energy expenditure, TEF: thermic effect of food

If energy intake exceeds in a sustained way total energy expenditure (TEE), excess energy will be stored in the form of triglycerides producing an adipose tissue expansion, which is linked to increases in lean mass and body weight. As a result, body weight might vary based on intake and TEE, which is determined by: resting energy expenditure (REE), exercise energy expenditure, thermic effect of feeding and thermogenesis energy expenditure [22, 23] (Fig. 2). REE is the minimum amount of energy an organism requires to be alive and represents between 55–75% of TEE. Most studies have determined that REE is higher in people living with obesity (PLWO) in contrast to those living with normal-weight. However, when body composition is considered, the effect of obesity is not entirely clear [23, 24]. Human metabolic rate can be evaluated by direct calorimetry or double-labeled water [25]. However, these techniques are very complex and are usually restricted to research purposes. In clinical practice, indirect calorimetry, which analyses oxygen consumed and carbon dioxide generated, represents the gold standard for REE assessment in this setting [26].

Adipose tissue is involved in many aspects of metabolism. It regulates energy expenditure, hunger, insulin sensitivity, bone metabolism, inflammation, immunity, reproductive and endocrine functions among others [11]. The mechanisms involved in adipose tissue dysfunction include hyperplasia and hypertrophy of adipose cells, hypoxia, pro-inflammatory state, impaired extracellular matrix remodeling and fibrosis, in association with an adipokine altered secretion [27]. The anatomical distribution of fat deposits determines their physiological roles and metabolic identity. Two main forms of adipose tissue can be identified: white and brown adipose tissue. Central obesity, defined as an enlarged white adipose tissue in the intra-abdominal area (visceral adipose tissue), is linked to metabolic risk, systemic low-grade inflammation, which is characterized by elevated C-reactive protein (CRP) levels and pro-inflammatory cytokines, insulin resistance (IR), and a higher risk of metabolic disorders prior to puberty [2, 27]. Adipose-derived proinflammatory cytokine secretion has been continuously related with the risk of adverse outcomes in obesity-linked complications, stimulating a consistent-low-grade inflammatory response [27]. Multiple adipokines as leptin, resistin, adiponectin, tumour necrosis factor α (TNF-α) and interleukin 6 (IL-6), among others, are involved in the regulation of inflammation and exhibit an important role in body homeostasis regulation [27–30].

Not surprisingly, inflammation markers are associated with adiposity measures and cardiometabolic risk [31]. Central obesity in children is independently associated with CRP, IL6 and TNF-α [32]. In the context of obesity, moderately elevated CRP levels are indicators of low-grade systemic inflammation and have been associated with the development of atherosclerosis, meta-inflammation, and increased body fat percentage (BF%) [33]. In children and adolescents, CRP levels are predictors of cardiovascular disease and can be frequently applied in clinical practice to categorize the coronary artery disease risk [34]. In children with obesity, chronic low-grade inflammation contributes to the appearance of early vascular changes [5]. Therefore, the adoption of healthy lifestyle changes in children to get lower CRP levels and a reduction of the cardiovascular risk is important [35].

The predisposition to obesity begins in intrauterine life. Several pregnancy-related factors contribute to childhood obesity [36] including maternal BMI, children of mothers with overweight or obesity during pregnancy have a 40% greater risk of developing obesity [37]. Other factors include physical inactivity during pregnancy, gestational diabetes [38], Cesarean-section delivery, and a high birth weight [39]. Conversely, infants born with a birth weight below 1500 g have a higher likelihood of developing central obesity [40].

In early childhood, additional risk factors emerge. The absence of breastfeeding in the first months of life [41], excess weight during early childhood [39], and early consumption of ultra-processed and hypercaloric foods all contribute to obesity risk. Notably, the intake of sugar sweetened beverages among children and adolescents (ages 3–19) has increased by 23% in the past 20 years, paralleling the rise in obesity prevalence [42]. Other contributing factors include low consumption of fruits and vegetables [36], disorganized eating patterns, such as skipping breakfast, which is linked to a higher incidence of childhood obesity, while eating more than three meals a day is associated with a lower risk of obesity [40]. Additionally, sedentary lifestyles [43], antibiotic exposure before the age of two [44], parental educational level [45], and inadequate sleep duration and quality [46] further increase the risk of childhood obesity.

Obesity associated with endocrinological disorders represents less than 1% of the causes of childhood obesity. Most children with high adiposity and endocrinologic diseases also have hypogonadism, short stature, and poor linear growth [47]. On the other hand, some pharmacological treatments can collaborate in the weight gaining process, for example glucocorticoids, antipsychotics (risperidone and olanzapine) and antiepileptics [48].

The present narrative review addresses the importance of childhood obesity, its correct diagnosis considering excess adiposity and its impact on health in this population at such an early age.

## Epidemiology

Obesity is a global epidemic, and its prevalence has tripled since 1975 [9, 49]. In 2016 around 340 million children and teenagers within 5–19 years of age were living with excess weight [9]. Later in 2020, 39 million children under 5 years of age had either overweight or obesity and almost 50% lived in Asia. In the United States (US), childhood overweight and obesity prevalence is 22.8% in children between 2–5 years, 34.2% in school-age (6–11 years) and 34.5% in teenagers (12–19 years) [47, 50]. According to recent reports of the European Union (EU), one in every 4 school-age children with obesity has severe obesity [51]. Other countries like South Korea, China, Indonesia, Thailand, Mexico and Latin America have also exhibited notable increases in childhood obesity prevalence [52–57]. According to the World Obesity Federation, approximately 206 million children and adolescents between 5–19 years will suffer from obesity in 2025 and 254 million in 2030 [40].

In the past, obesity was considered a problem only in first world countries. However, Weber et al. in their meta-analysis suggested that among white, Hispanic, and other ethnic populations living in the US, those with low economic incomes were more likely to have overweight or obesity compared to those with medium or high incomes [58]. In addition, a significant increase has been observed in developing countries, particularly in the urban areas [9].

It is well known that the prevalence in adulthood is influenced by:

i. Child´s age; most adolescents with obesity will suffer from this condition during adulthood [47, 51].
ii. Severity of obesity, around 71% of adolescents with severe obesity will remain in the obesity range in adulthood [47].
iii. Presence of overweight or obesity in one or both parents; obesity in parents doubles the risk of obesity in adulthood, predominantly in children under 10 years [47].

The SARS-CoV2 pandemic further contributed to an increase in body weight among children and adolescents due to a decrease in physical activity, excessive use of screens, diet changes as well as increased family and individual stress [59].

## Impact on health

Childhood obesity is related to the appearance of cardiovascular disease in adulthood [5]. In 2017 pediatric patients with overweight and obesity reportedly exhibited a higher risk of adiposity and worse scores on cardiovascular risk scales [60]. In the same year, it was determined that childhood obesity could be a risk factor for some cardiovascular risk variables in adults and highlighted that childhood obesity has a positive and significant correlation with the development of hypertension (HT) and hypertriglyceridemia in adulthood [61]. A high percentage of fat mass and BMI at 10 years of age was observed to correlate with elevated systolic (SBP) and diastolic blood pressure (DBP), with an increase in VLDL, LDL, triglycerides, and insulin levels at 18 years old [62]. Furthermore, it was shown that an elevated BMI in children is associated with poor cardiovascular health, elevated BP, and increased left ventricular mass index [63, 64].

**Fig. 3:**
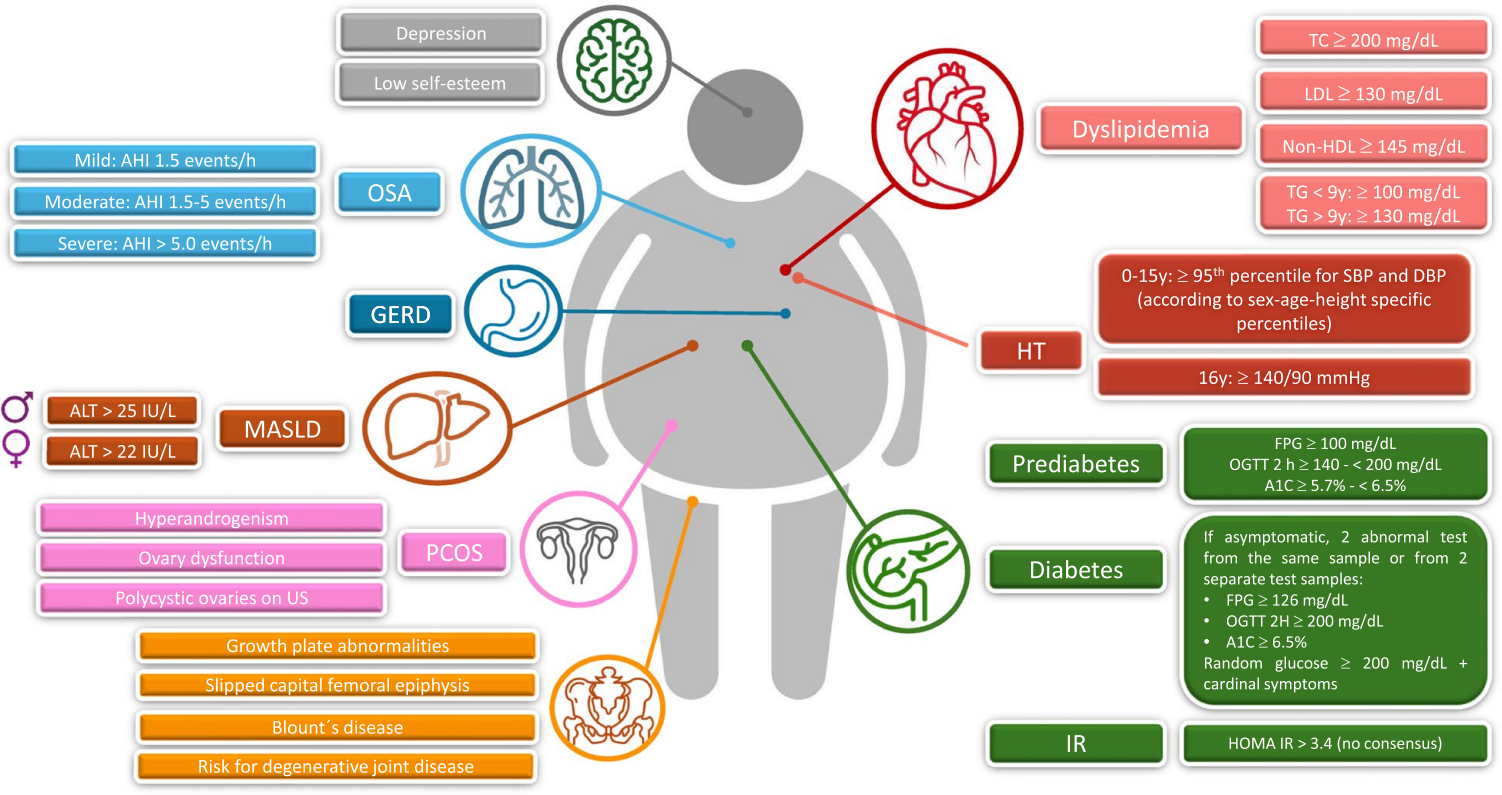
Childhood obesity comorbidities. Children and adolescents with overweight and obesity have a high risk of developing multiple comorbidities such as IR, T2D, dyslipidemia, HT, coronary disease, among others during their adulthood, which increases the morbidity and mortality of these patients. The high prevalence of this comorbidities associated with overweight, or obesity emphasizes the importance of early interventions to prevent unfavorable outcomes. AHI: apnea hypopnea index, DBP: diastolic blood pressure, FPG: fasting plasma glucose, GERD: gastroesophageal reflux disease, HT: hypertension, IR: insulin resistance, LDL: low density lipoproteins, MASLD: metabolic dysfunction-associated steatotic liver disease, Non-HDL: Non-high density lipoproteins, OGTT: oral glucose tolerance test, OSA: obstructive sleep apnea. PCOS: polycystic ovary syndrome, SBP: systolic blood pressure, T2D: type 2 diabetes, TC: total cholesterol, TG: triglycerides, US: ultrasound

Interestingly, it has been shown that children with a consistently high BMI throughout childhood had a lower cardiometabolic risk compared with young people who developed a significant increase in adiposity in late adolescence [65]. A predominantly elevated BMI before puberty results in an increased prevalence of type 2 diabetes (T2D), HT, and dyslipidemia compared to normal weight peers [66]. Thus, the goal should be to achieve a normal body weight range before the onset of puberty and, consequently, a slowdown in the progression of T2D development, cardiovascular disease, and obesity-related cancer during adulthood [67] (Fig. 3).

### Metabolic syndrome

**Fig. 4:**
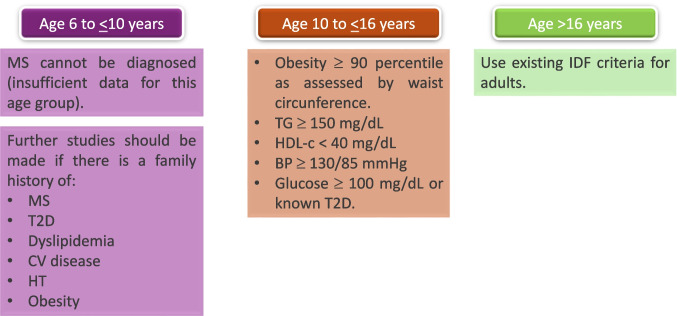
Metabolic syndrome definition in pediatric population according to the IDF criteria. Metabolic syndrome (MS) increases the risk of cardiovascular disease. Children and adolescents with MS have at least 3 of the following components: high blood pressure, abnormal basal glucose levels, low HDL, high triglycerides or high abdominal circumference. BP: blood pressure, CV: cardiovascular disease, HDL-c: high density lipoproteins, HT: hypertension, IDF: International Diabetes Federation, T2D: type 2 diabetes, TG: triglycerides

Childhood obesity is closely associated with the metabolic syndrome (MS). Its prevalence rate in adolescents in the US varies from 2.0% to 9.8% in all youth and from 12.4% to 44.2% in adolescents with obesity [68, 69]. Children with obesity have 5 times greater risk of suffering obesity in adulthood [50, 69]. Adiposity in children is related to the progression of MS in adulthood [70]. MS increases the risk of cardiovascular disease by 1.5 to 2 times in adults and children of all ages [71], being more prevalent in children with severe obesity [72, 73]. Whilst the MS definition is well established in the adult population, there is no internationally accepted definition in pediatric patients [74, 75]. The MS was defined as a combination of 3 of the following elements: abdominal circumference or BP above the 90th percentile, basal plasma glucose > 110 mg/dL, triglycerides > 110 mg/dL, and HDL cholesterol < 40 mg/dL [76]. The International Diabetes Federation (IDF) published its MS definition in 2007 [77]. It concluded that children older than 10 years who met 3 of the following criteria could be diagnosed of MS: elevated waist circumference (WC), HT, IR, and dyslipidemia. The IDF recommended the use of different cut-off points for each parameter based on each age group. However, this consensus also established that children younger than 10 years could not be diagnosed of MS due to the absence of specific reference values for those ages (Fig. 4). Subsequently, a quantitative scale for MS in children between 2–11 years, using anthropometric and metabolic parameters according to age and sex was proposed [78]. Currently, to optimize the stratification of children at risk for MS, strict monitoring is recommended in those with a BMI above the 90th percentile, while an urgent intervention should be performed in those with a BMI above the 95th percentile [78].

### Alterations in glucose metabolism

Dysglycemia is a term used to describe high blood glucose levels, and it includes prediabetes, impaired glucose tolerance (IGT) and T2D [79]. Children develop complications and clinical manifestations of diabetes in a more rapid and aggressive way than the adult population [32]. Obesity throughout adolescence has also additionally been related with a higher risk of mortality from T2D and cardiovascular disease during adulthood [80]. For years adolescents with T2D have had a poor therapeutic response and a poor glycemic control with available treatment regimens [81, 82], with an early-onset T2D with prolonged exposure to metabolic disturbances amplifying micro- and macrovascular long-term complications [83]. The advent of glucagon-like peptide 1 receptor agonist-based therapies, such as Liraglutide and Semaglutide, for diabetes and obesity management seem to represent a turning point in those scenarios, with a BMI reduction of approximately 5% to 17% with 1 year of treatment [80, 84, 85]. The most significant effect was observed with Semaglutide, with a BMI decrease of 6.0 points after 16 months [86].

### Insulin resistance

IR is the earliest metabolic abnormality in people with alterations in glucose metabolism and is present before the onset of T2D. IR is defined as a condition in which normal plasma insulin concentrations have lost their ability to promote adequate peripheral glucose deposition, to suppress hepatic gluconeogenesis and to inhibit the production of LDL molecules [87]. Hepatic IR leads to a decrease in triglyceride production, whereas peripheral IR causes a worsening of glucose tolerance [88].

Ectopic fat deposition acts as a potential cause of IR. The adipose expansion hypothesis (allostatic theory) suggests that after exceeding the adipose tissue storage capacity there is an increase in the flow of lipids to non-adipose tissues, which causes lipotoxicity and leads to IR [89]. Lipid storage within insulin-sensitive tissues, such as musculoskeletal, hepatic, pancreas, and adipose tissue, is related with established IR in molecular pathways associated to glucose metabolism, producing hyperinsulinism [90, 91]. In people with obesity, the antilipolytic actions of insulin in adipocytes together with an impaired lipid storage within these cells, leads to a rise in free fatty acids flux, systemic inflammation, and lipid accumulation in non-adipose tissues, triggering an increase in IR [92, 93]. The systemic response to IR associated to alterations in lipid metabolism manifests as dysglycemia and alterations in cardiovascular biomarkers like high concentrations of triglycerides, VLDL and inflammation markers, with low levels of HDL and adiponectin [94, 95].

A discrete weight loss has beneficial effects for insulin sensitivity. A decrease of 0.30 standard deviations (SD) of BMI in adolescents with obesity is linked to a substantial reduction in intrahepatic and intramuscular fat deposition, with an increase in insulin sensitivity [96, 97].

A greater benefit has been observed if a BMI reduction higher than 0.50 SD is achieved [98, 99].

IR assessment allows an early intervention to avoid or delay the diabetes onset [100]. It is difficult to have a single biochemical IR definition in the pediatric population since there is no globally accepted numerical definition. The gold standard for measuring IR is the euglycemic hyperinsulinemic clamp, however, its high complexity and cost have discouraged its use in daily clinical practice [101]. For this reason, other models have been established to estimate the degree of IR such as Homeostasis Model Assessment of Insulin Resistance (the HOMA-IR), validated in children and adolescents and the Matsuda Insulin Sensitivity Index (ISI), among others [102].

The HOMA-IR is the most used method in the pediatric population [103, 104]. It estimates beta-cell function and insulin sensitivity, using basal plasma glucose and insulin values. Some studies have established a value of 3.43 as the "optimal cut-off point" to determine those people at risk of developing cardiovascular disease [105]. Insulin sensitivity measured with the euglycemic hyperinsulinemic clamp has a strong correlation with the HOMA-IR [106]. The first and second phase of insulin secretion correlate with basal insulin levels and the HOMA-IR. The (OGTT) was validated for its use in the pediatric population, with a high correlation with the euglycemic hyperinsulinemic clamp [107]. The Matsuda Index has showed a higher sensitivity for the detection of metabolic disturbances in children with obesity in contrast to the HOMA-IR [108]. A better correlation of the Matsuda Index with the euglycemic clamp than with the HOMA-IR has been reported [109]. One explanation could be that the HOMA-IR may be affected by nocturnal GH secretion, which affects insulin secretion, whereas, the Matsuda Index may eliminate this interference, assessing the immediate response of glucose and insulin metabolism.

Puberty is associated with a decrease in insulin sensitivity in youth with normal-weight or obesity, predominantly in females [110]. Under normal conditions, IR is resolved after the completion of puberty, however, this is not the case when obesity coexists. Interestingly, patients with obesity have a higher IR during puberty in contrast to their peers with weight in the normal range.

### Type 2 Diabetes

Obesity in pediatric patients is related to a higher risk of prediabetes and T2D. The prediabetes definition includes a fasting plasma glucose (FPG) ≥ 100 mg/dL and < 126 mg/dL, a glucose at 2 h after an OGTT ≥ 140 mg/dL and < 200 mg/dL or a glycated hemoglobin (HbA1c) ≥ 5.7 and < 6.5%.Diabetes is defined as a FPG ≥ 126 mg/dL, glucose at 2 h after OGTT ≥ 200 mg/dL, HbA1c ≥ 6.5%, or a random blood glucose ≥ 200 mg/dL associated with fatigue, polyuria, polydipsia, and weight loss [111]. It should be noted that the cut-off points for prediabetes and diabetes diagnosis are like those used in adults [112].

The American Diabetes Association (ADA) and the International Pediatric Diabetes Society recommend a prediabetes and diabetes screening in children and teenagers with obesity who have a maternal history of diabetes or gestational diabetes, diabetes family history signs, or conditions compatible with IR (acanthosis nigricans, HT, dyslipidemia, polycystic ovary syndrome), or history of small for gestational age [113]. Screening tests should include FPG, OGTT and HbA1c. Biochemical follow-up is recommended at least every 3 years if the results are normal [111, 113].

An early onset of T2D is associated with a longer duration and more rapid progression of hyperglycemia [114]. Young men with overweight have a threefold increased risk of developing T2D before the age of 25 years, whereas adolescents with obesity have a significantly 27-fold increased risk [115]. Females are more affected than males if they have a high BMI during adolescence, with a sixfold increased risk of developing T2D in adolescents with overweight and a 45-fold increased risk if they have obesity [115]. Severe obesity seems to be the main risk factor for T2D in the pediatric population. About one third of children with T2D have been found to have a BMI > 40 kg/m^2^ and an additional 17% possess a BMI > 45 kg/m^2^ [116].

### Dyslipidemia

Dyslipidemia in children with overweight or obesity leads to alterations in the function and structure of the vascular system because of its association with high VLDL levels and a subendothelial retention of LDL-containing molecules [117]. Atherosclerosis begins in childhood and has a positive association with BMI, age, triglyceride and LDL concentrations, and an inverse association with HDL levels [118]. Noteworthy, the amount of total cholesterol in childhood positively predicted carotid intima-media layer thickness, with BMI during childhood predicting its thickness in the female population [119]. The International Childhood Cardiovascular Cohort Consortium conducted a meta-analysis where the number of cardiovascular risk factors during childhood (hypercholesterolemia, hypertriglyceridemia, HT, and high BMI) was a predictor of progressive thickening of the carotid intima-media layer during adolescence [120]. It is important to monitor lipid levels in children with overweight or obesity since alterations in lipid metabolism could be identified at early stages [121]. Moreover, a slight weight loss is related with a significant decrease in triglyceride levels and an increased in HDL concentrations, reducing the cardiometabolic risk [121].

### Hypertension

HT in the pediatric population is determined by a systolic and diastolic blood pressure (SBP and/or DBP) greater than or equal to the 95th percentile according to age, sex, and height, at least in 3 different measurements [122]. Among children with obesity, the prevalence of HT is as high as 30%, in contrast to less than 5% in those within the normal-weight range [123]. People who have an increase in adiposity during childhood and early adulthood exhibit a 3.7-fold increased risk of developing HT throughout adulthood compared with those with a BMI within the normal-weight category [124]. People with overweight or obesity who achieve the normal weight range in early adulthood do not have an increased risk of HT in the middle age [124].

The pathophysiology of HT in childhood is complex and includes several systems [5]. These pathways involve the sympathetic nervous system (SNS), elevated cortisol, adipokines, oxidative stress, and inflammation [125, 126]. The presence of a high BF% is linked with increased activation of the renin–angiotensin–aldosterone system and sympathetic tone [127]. This hyperdynamic circulation results in left ventricular (LV) dilatation and hypertrophy [128]. Obesity and HT lead to an increase in peripheral vascular resistance producing hyperdynamic circulation. These modifications can trigger changes and remodeling of large caliber arteries and myocardial structure [74]. HT during adolescence is a significant endothelial dysfunction predictor, therefore, an adequate BP assessment and follow-up is essential.

### Metabolic dysfunction-associated steatotic liver disease (MASLD)

MASLD is a multifactorial and complex entity, it involves an interaction between genetic, epigenetic, and environmental factors and coexists very frequently with risk factors of MS and T2D [129, 130]. A high prevalence of MASLD has been observed in Hispanic children, while a reduced prevalence of this condition in young African American is reported [131, 132]. Childhood obesity contributes to an increased risk of MASLD later in life [133]. The presence of obesity as early as at 2 years of age may influence the development of MASLD in adolescence, with a ninefold increased risk among children with obesity at 5 years of age in contrast with their peers with a BMI within the normal-weight category [134]. Adiposity is a significant risk factor for MASLD, with a clear association between adiposity in childhood and adverse liver events in adolescence, making it the most frequent cause of chronic liver disease in children in Western countries [135, 136]. The adiposity-related risk is predominantly attributed to IR and adipose tissue dysfunction in these patients. The presence of MASLD in the pediatric population can predict the premature development of chronic disease with a more aggressive course, specifically once steatohepatitis is already established [137, 138]. Noteworthy, MASLD increases the risk of cirrhosis and hepatocarcinoma, and contributes to the development of T2D, cardiovascular and renal disease.

Children with MASLD are at increased risk of presenting biochemical alterations compatible with IR, elevated basal glycemia, HbA1c, basal insulin and HOMA-IR [139]. The presence of T2D increases the metabolic liver disease risk by twofold compared with people without diabetes, whereas MASLD is associated with a 2.5-fold increased risk of developing T2D [140]. IR and T2D are related with more advanced stages of MASLD [137, 141].

According to the clinical guidelines of ESPGHAN and NASPGHAN (European and North America Societies for Pediatric Gastroenterology, Hepatology and Nutrition), screening for MASLD is recommended in pediatric patients with a BMI above the 95th percentile. The NASPGHAN recommends an ALT measurement in all children with overweight and obesity over 9 years of age [142, 143] and an abdominal ultrasound. The screening main goal is an early detection of liver disease in children and adolescents with high-risk factors [144].

## Diagnosis

There is controversy about which is the ideal indicator for measuring overweight and obesity. Currently, BMI is used as a standardized measure for measuring either of these 2 conditions. However, the BMI may miss those cases in which body composition is pathological despite a BMI within the normal-weight category [145, 146].

Body composition in children changes progressively, reflecting dynamic differences in fat mass (FM) and fat-free mass (FFM) [147]. After birth, rapid weight and body composition modifications occur. At 6 months of life the total body fat percentage increases around 30%. At 2 years of age, it decreases to an average of 19.5% in males and 20.4% in females, while at 5 years of age the BF% is around 14.6% in males and 16.7% in females [148]. At the end of childhood, females begin to accumulate more fat than males, while males gain slightly more FFM compared to females [149]. During prepuberty (Tanner stage I), females have a higher BF% than males, with a variation between 1–3%. Throughout puberty (Tanner stages II-III), females accumulate 4–6 kg more FM compared to males [150, 151]. It is of great importance to take these differences into account when pediatric patients are evaluated.

**Fig. 5:**
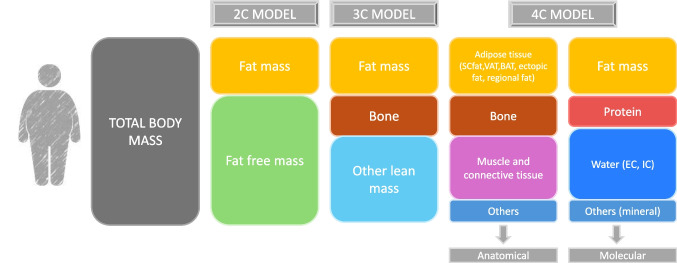
Body composition components in the 2C, 3C and 4C (multicompartmental) models. Different techniques are available to evaluate the body composition in children and adolescents, each one of them with its advantages and limitations. The body is divided into numerous compartments for the body composition assessment in the different models. The 2-compartment model (2C-Model) separates the body into fat-free mass and fat mass; this model is widely used in clinical practice because of its simplicity and easy use. The 3-compartment model (3C-Model) divides the body into fat-free mass, fat mass and bone; children´s body composition has been evaluated employing this technique. In addition, the gold standard, the 4-compartment model (4-Model) expands the previous compartments to fat mass, protein, total body water and bone; due to the possibility of extracting the protein and mineral content from the fat-free mass, the most precise measurement between the available techniques is guaranteed. 2-C Model: 2 compartment model, 3-C Model: 3 compartment model, 4-C Model: 4 compartment model, BAT: brown adipose tissue, EC: extracellular, IC: intracellular, SC: subcutaneous, VAT: visceral adipose tissue

Different approaches have been used for the estimation or direct measurement of body composition. The selection of the measurement method relies on which compartments are of interest, the degree of precision needed, and if the body composition evaluation is required for investigation or clinical purposes [152]. Available methods include anthropometry, electrical bioimpedance, magnetic resonance imaging (MRI), hydrometry, air displacement plethysmography (ADP), ultrasonography, and dual X-ray absorptiometry (DXA) [153]. The body composition study models can divide the body in different compartments: the 2-compartment model (2-C model) divides the body into FFM and FM, this model is applied in clinical practice because of its simplicity and easy use. Nevertheless, it is subject to error due to the presumption that FM and FFM composition does not change over the years or with disease states [152]. The 3-compartment model (3-C model) divides the body into FFM, FM and bone. The 4-compartment (4-C model) or multicompartmental model divides the body according to its anatomical or molecular composition with this model being mainly applicable in research settings [152]. With the 4-C model it is possible to extract the protein and mineral content from the FFM, guaranteeing the most precise measurement between the available techniques [154]. Another benefit of employing this model in children is the removal of the presumption of a static proportion of the relevant tissue content in the body (Fig. 5). As children and adolescents grow up, there are critical changes in the body composition, which should be considered [155]. Nowadays, there are no standardized cut-off points for determining excess body fat in pediatric patients. However, many studies support the utilization of the same cut-off points for obesity as in the adult population (25% in males and 35% in females) [146, 151, 156]. Different values have been proposed, as the International Obesity Task Force (IOTF) body fat cut-off points or the age-specific and gender-specific 85th or 95th percentile. Nonetheless, it is not established whether these definitions lead to similar results with respect to the prevalence of pediatric obesity [157].

### Simple anthropometric measurements

#### Body mass index (BMI)

In clinical practice, obesity is determined by the BMI, which is a practical tool but not a good body composition estimator. BMI results from dividing weight (in kilograms) and height (in meters^2^) [158]. In 2000, Cole et al. [159] published BMI cut-off points for overweight and obesity according to sex, at ages 2 to 18 years, which are widely accepted in the adult population [159]. This study provided a less arbitrary and more international definition compared to others. Nevertheless, this BMI classification is not absolved of a misclassification of overweight and obesity when it is compared to BF%. In a recent study from our group, approximately 22% of children and adolescents with normal weight according to BMI had overweight and obesity according to their BF% [146].

#### Skinfold thickness (ST)

ST involves measuring the thickness of 2 layers of subcutaneous (SC) adipose tissue with a caliper for body fat in selected body sites, the most used are biceps and triceps, subcapsullar, supraumbilical and iliac crest [154]. ST is frequently used in the pediatric population because it is a non-invasive, reproducible and inexpensive method. It is considered together with the WC and bioelectrical impedance analysis (BIA) as a double indirect or predictive method, in which an indirect technique is utilized to validate a generalized prediction of body composition from another measurement [160]. There are many limitations of the technique as the high variability between observers, wide range of body fat calipers, variations in the measurement site chosen and the need to apply body composition formulas which restrict its application to the population for which they were designed [155, 161, 162]. Some studies have evaluated the correlation among the results determined from body water, body density, bone mineral content measurements and eight widely used skinfold thickness Eqs. [163, 164]. The main result was an over or underestimation of FM using skinfold measurement, of approximately 10%.

### Equipment-based methodologies

**Fig. 6:**
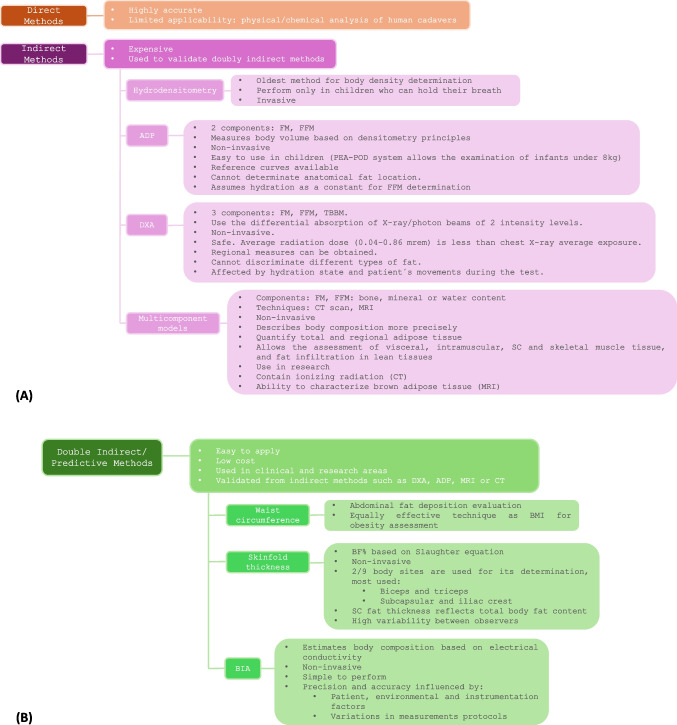
Body composition techniques (direct and indirect methods). Over the years, different body composition measuring methods have been developed. Body composition was quantified by dissecting cadavers, which was considered a direct method. Subsequently, new methods were design to measure body composition indirectly, such as hydrodensitometry, air displacement plethysmography (ADP), dual energy X ray absorptiometry (DXA), CT-scan, MRI and other indirect measuring methods as bioelectrical impedance analysis (BIA) and anthropometrics. The 4-compartment model (4-C Model), ADP and DXA are used as reference methods in adult and pediatric population. ADP: air displacement plethysmography, BF: body fat, BIA: bioelectrical impedance analysis, BMI: body mass index, CT: computed tomography, DXA: dual energy X ray absorptiometry, FFM: fat-free mass, FM: fat mass

Several techniques are currently available to evaluate and quantify body composition (Fig. 6). One of the most widely used is the bioelectrical impedance analysis (BIA), which estimates FM and FFM using specific equations for the device and reference population [165]. More sophisticated techniques are used in the research area, including ADP, DXA, computed tomography (CT-Scan), MRI and other multicompartmental models [166].

### Bioelectrical impedance analysis (BIA)

The basis of BIA is the difference in the electrical impedance of different tissues (e.g. FM and FFM), in response to an electrical current flowing at a particular frequency. Due to their dissolved electrolytes, the body´s lean tissues are the primary electrical current conductors, whereas bone and body fat are comparatively weak conductors [152]. BIA estimates total body water and, by assuming a constant hydration, determines FFM. Body lean mass is determined from total body water, considering that in adults 73% of the FFM is composed of water. Nevertheless, the 73.2% reference factor for the FFM water content causes a FFM overestimation and FM underestimation in children who have 75–76% water for the FFM. Consequently, variations in the hydration status are the biggest limitation of this technique [167]. The main BIA benefits are its simplicity, quick analysis and reasonably priced equipment. To guarantee reliability and consistency, each study needs to be carried out under specific conditions; patients should avoid vigorous physical activity 12 h prior to the test, be adequately hydrated and preferably should remain on an empty stomach. In addition, the patient must wear light clothing and should not wear jewelry or body cream before the test [168]. The use of the same device is recommended to carry out body composition studies [169]. BIA results are affected by adiposity degree, hydration state, skin temperature, size and sex. Differences in the length of the limbs and trunk can affect the association between total body water and FFM, therefore, mathematical estimation model validation against a reference method is suggested, in addition to adjustment according to age and sex [170].

### Air Displacement Plethysmography (ADP)

ADP is a validated and reproducible alternative to hydrodensitometry, which is viewed by some as the gold standard for body composition determination [171]. ADP can be a reliable method for measuring body fat in children and adolescents when standard procedures and specific equations are followed [172]. It uses the pressure/volume ratio to estimate the volume and with this value and the weight of the person calibrates the density to indicate the BF%. This bicompartmental technique has been shown to predict the amount of FM and FFM very accurately [171]. PEA POD® and BOD-POD® are the registered commercial names of the body composition analysis devices that use the ADP technology. The PEA POD® is the machine used for infants weighing between 1 and 8 kg, while the BOD-POD® is the equipment used for adults and children. ADP is frequently employed in infants (under 6 months) applying the PEA POD® whereas in children (above 6 years old) and adults the BOD-POD® is used [173–176]. Infants are placed in an air chamber to determine the displaced volume after weight is measured on a scale. The measured mass and volume are used to calculate the density, which is then used in a predictive equation to estimate the infant´s FM and FFM, which are then employed to determine the BF% [177]. ADP has multiple advantages, among which is the radiation absence, non-invasiveness, measurement speed [178, 179] and validation for its use in infants. When using child-specific lung volume equations, the ADP accuracy and precision are comparable to the 4-C model in school-age children [152]. Comparison of ADP with hydrostatic weighing, which is the reference method for body volume measurement, has demonstrated significant agreement in the children´s body composition assessment [180, 181]. Likewise, when contrasting the results obtained through ADP with those of the DXA, a substantial association in the BF% was observed [182]. Nevertheless, ADP is not a perfect technique, among its disadvantages is the high cost of the test, the need to undergo through multiple calibration rounds before the patient´s evaluation and the assumption of hydration as a constant for the FFM determination [170, 179]. According to studies, children and adolescents are more hydrated than adults, leading to a FFM overestimation by ADP and BIA and a reduction in FFM density and specific resistivity [167, 177]. FFM hydration changes with weight status, with higher hydration levels in patients suffering from obesity in comparison with those without this condition [183]. ADP considers the assumption of consistency of lean body mass attributes, which vary with age and physical maturity, as well as in states of overhydration or dehydration, which may considerably affect the result [155]. Along with the DXA, one of the most popular methods for body composition determination in research is the ADP [184].

### Dual-energy X-ray Absorptiometry (DXA)

The body composition assessment via DXA depends on variations in the X-ray absorption degree by different groups of tissues [178]. DXA has become the method of choice in the clinical setting when it comes to body composition analysis [185], allowing to obtain FM, FFM, bone and soft tissue contents [186]; which can be useful in clinical practice and research settings, despite the lack of normative values for regional assessments [172, 184]. DXA is becoming more accessible in the clinic and is being used to determine the children and adolescent´s body composition [187–189]. The current equipments permit the evaluation of body composition with a single whole-body scan, providing faster results and less radiation exposure [190]. DXA is a safe, rapid and non-invasive method with a high precision and accuracy. It requires the use of X-rays at very low doses that are harmless, even in children [191]. The average radiation dose is smaller than a chest radiography, ranging from 0.04 to 0.86 mRem, depending on the body size and device [192]. DXA cannot differentiate different soft tissue types as muscle or internal organs [193]. Other DXA limitations are the requirement for trained personnel, the high cost of the test [179] and the exposure to minimal cumulative radiation levels if frequent follow-up is performed. Institutional review boards suggest limiting DXA scans to twice a year and prohibit the use of this technique in infants under 3 months [153].

### Ultrasonography

Ultrasound has emerged as a valuable tool for assessing body composition in children and adolescents, in the context of obesity [194]. This non-invasive, radiation-free technique enables precise measurement of SC and visceral fat, as well as muscle thickness. Studies have demonstrated that ultrasound measurements of SC adipose tissue correlate strongly with BMI and WC, providing a reliable assessment of adiposity in pediatric populations. Moreover, ultrasound has been shown to accurately quantify intra-abdominal fat, offering insights into obesity-related health risks. Its portability and safety make ultrasound a practical choice for routine evaluation and monitoring of body composition in clinical and research settings involving children and adolescents [195].

### Computed Tomography (CT-Scan)

CT scan evaluates SC, intramuscular, skeletal muscle tissue as well as the amount of fat infiltrating lean areas [162, 196, 197]. Imaging techniques are considered the gold standard for measuring intra-abdominal fat [198]. CT estimates visceral fat and provides an accurate assessment of hepatic and muscular fat content [199]. The anatomical area frequently utilized as a reference is situated 5 cm above the transition between the fourth and fifth lumbar vertebra [162, 197]. This technique has many advantages, such as high precision, high image resolution and the ability to determine tissue quality [200]. Although validated cut-off points have been established for adults [200], no universally accepted cut-off points have been validated for children and adolescents, despite some studies exploring this area [201]. CT disadvantages are its high cost, exposure to radiation, which makes this technique inappropriate for children´s follow up, lack of portability of the equipment and requirement of technical skills for image analysis [200].

### Magnetic Resonance Imaging (MRI)

MRI is a technique that is used more frequently in children and adolescents [202], it evaluates tissue density from the cross-sectional images obtained [179]. MRI is an accurate 4-C model and evaluates skeletal muscle mass, lean mass and FM [179]. MRI is accepted as a gold standard for children´s body composition determination and for quantitative assessment of visceral adipose tissue and has been employed to generate precise predictive equations for FM [203]. MRI can assess body composition at the tissue and organ level by quantifying the target volume and adding the tissue´s and organ´s cross-sectional areas acquired in cross-sectional images [179]. It has been demonstrated that MRI is a reliable and accurate non-invasive technique for determining body composition [152]. MRI does not utilize ionizing radiation, and the results are not dependent on FFM hydration [152]. In addition, MRI has the capacity to morphologically characterize brown adipose tissue, especially in children and adolescents [179]. In a study conducted on pediatric population, MRI showed a strong association with ADP results [204] and has shown to have comparable accuracy to CT in quantifying SC and visceral adipose tissue in adolescents and adults with obesity [205]. However, in other studies MRI seems to underestimate FM in people with higher adiposity levels [206]. Among its disadvantages are the elevated cost, the requirement for sedation in some cases, the time required to perform the test and the need for sophisticated equipment and a skilled team [179].

### Hydrodensitometry (HW- hydrostatic weighing)

HW calculates the person´s body volume via the Arquimides´ principle and using body density to evaluate the proportion of FM and FFM [178]. This technique has originally been established as the gold standard for body composition measurement. Nowadays, it is mostly used in the research area as a reference method for body composition determination, particularly to validate other techniques [180, 207, 208]. HW disadvantages are the cumbersome and extended duration (75 min), the requirement for extra preparation and the patient´s complete submersion in water, which is an important obstacle for its use in younger patients [207]. Only children who can hold their breath for a prolonged time and those who do not have contraindication for underwater submersion can participate in HW. Additionally, this technique is inaccurate in younger children due to the variations in lean body mass density throughout maturation [209].

## Conclusions

Obesity is a chronic disease that has become a worldwide epidemic across all age groups, with an alarming increase in the child population. Accurate diagnosis is crucial for early intervention, and while traditional methods such as BMI and WC remain widely used, advanced diagnostic tools—including ADP or imaging techniques like ultrasound, CT, and MRI—offer a more precise assessment of body composition and fat distribution. An adequate understanding of the etiology, pathophysiology, and the most appropriate diagnostic methods will enable health professionals to better manage this multifactorial and complex disease, ultimately helping to mitigate its associated cardiometabolic risks and long-term prognosis.

## Data Availability

No datasets were generated or analysed during the current study.
